# Ghrelin and Leptin among Patients with Urolithiasis with Concomitant Hyperuricemia and Metabolic Syndrome

**DOI:** 10.3390/biomedicines11020285

**Published:** 2023-01-19

**Authors:** Michalina Lubawy, Anna Blacha, Marcin Nowicki, Tomasz Deja, Krzysztof Wałkowski, Dorota Formanowicz

**Affiliations:** 1 Department of Medical Chemistry and Laboratory Medicine, Poznan University of Medical Sciences, 60-806 Poznan, Poland; 2 Ministry of Internal Affairs Hospital Poznan, Dojazd 34, 60-631 Poznan, Poland

**Keywords:** urolithiasis, hyperuricemia, metabolic syndrome, leptin, ghrelin, inflammation

## Abstract

**Introduction**: The study evaluated the selected appetite hormones (ghrelin, leptin) and inflammatory parameters (tumor necrosis factor alpha (TNF-α) and interleukin 6 (IL-6)) in patients with urolithiasis, metabolic syndrome (MetS), and hyperuricemia. **Materials:** 57 patients with urolithiasis, MetS and hyperuricemia (UP group) and 29 healthy people as the control group (CG group) were recruited to the study. All persons were 22–60 age. **Methods:** After preliminary testing, the qualified participants were evaluated for fasting serum levels of ghrelin, leptin, IL-6, and TNF-α. **Results:** Our results revealed differences between average values of leptin (*p* = 0.045), ghrelin (*p* < 0.001), IL-6 (*p* < 0.001), and TNF-α (*p* < 0.001) in the studied groups. Moreover, in the UP group, significant correlations were found between ghrelin and leptin; between these hormones and IL-6, and between leptin and uric acid (UA). Besides, leptin concentration increased significantly along with the changes in the body mass index (BMI) categories in the UP group. **Conclusions:** This study showed that patients with urolithiasis, concomitant MetS, and high UA levels may have problems managing appetite hormones.

## 1. Introduction

Urolithiasis has become an increasingly common disease in recent decades, along with the increasing trend of obesity, impaired glucose tolerance, and diabetic conditions. Its prevalence is estimated at 5–15%, depending on the region [1,2]. It has been found that kidney stones can consist of up to 100 different chemical compounds [2,3], and there are many reasons for their formation.

Our study has focused on uric acid (2,6,8-trioxypurine, UA), an organic chemical compound forming stones. It exists in serum as urate, the salt of UA, and varies significantly with diet. The ingestion of purines, endocrine production, and excretion drive the balance of UA levels [4].

UA is the final metabolite of endogenous and exogenous purine [5]. It cannot be catabolized to allantoin in primates and humans because of the evolutionary loss of urate oxidase. Its amount depends primarily on endogenous synthesis and the type of food consumed [5,6]. A purine-rich diet, increased purine metabolism, and excessive alcohol consumption, lead to increased UA synthesis. Moreover, tumor lysis syndrome, in which large numbers of cells are damaged, and nucleic acid metabolism is stimulated, also leads to increased UA production [7]. The vast majority of UA is excreted through the kidneys (65–75%) and intestines (25–35%) [8].

The imbalance between UA production and excretion leads to hyperuricemia, which is defined as serum concentration of UA in men and post-menopausal women, above 7 mg/dL (416.4 µmol/L) and above 6 mg/dL (356.9 µmol/L) among pre-menopausal women [8,9,10].

UA is both a pro-oxidant and an antioxidant. In the extracellular environment, it is considered an antioxidant that protects the erythrocyte membrane against lipid oxidation [8]. It is a potent-free radicals scavenger accounting for half of the antioxidant capacity of human plasma [11]. The increase in the level of UA in the serum may be a reaction of the body to the harmful effects of excessive oxidative stress, proven in cardio-metabolic and vascular diseases. On the other hand, cellular studies have demonstrated that UA may also exert pro-oxidant effects depending on its chemical microenvironment. Moreover, in the intracellular environment, it has a proinflammatory impact [11], inducing the NF-κB signaling pathway. NF-κB is a protein complex that controls the transcription of several cytokines and inflammatory molecules [12].

A solubility study showed that serum was supersaturated for monosodium urate crystal when the concentration of UA exceeded 6.5 mg/dL (386.6 µmol/L) [13]. Consequently, UA and urate crystals may deposit in the joints, kidneys, and other tissues, inducing tissue injury.

UA stones are the third most common type of kidney stone in the industrialized world. They are mainly formed due to acidic urine; less decisive factors are hyperuricosuria and low urine volume, which may be genetic, secondary, or idiopathic [14]. Increased excretion of UA is generally insufficient to adversely increase urine acidity, although it is involved in the broader process leading to this effect. However, defective ammonia buffering caused by reduced ammonia production in the kidneys further lowers urine pH, causing UA stones to form. There is a relationship between impaired ammonia and metabolic syndrome (MetS). It is most likely related to insulin resistance, which leads to impaired excretion of UA at low urinary pH, contributing to the formation of urate stones [15,16]. Each component of the MetS is also an independent risk factor for more acidic urine. Despite the great interest in the mechanism of obesity and kidney stones, many questions are still open in this debate, and one is the significance of various adipokines. One can suggest that ghrelin and adipokines, including leptin, may be related to blood UA levels [17,18,19].

**Figure 1:**
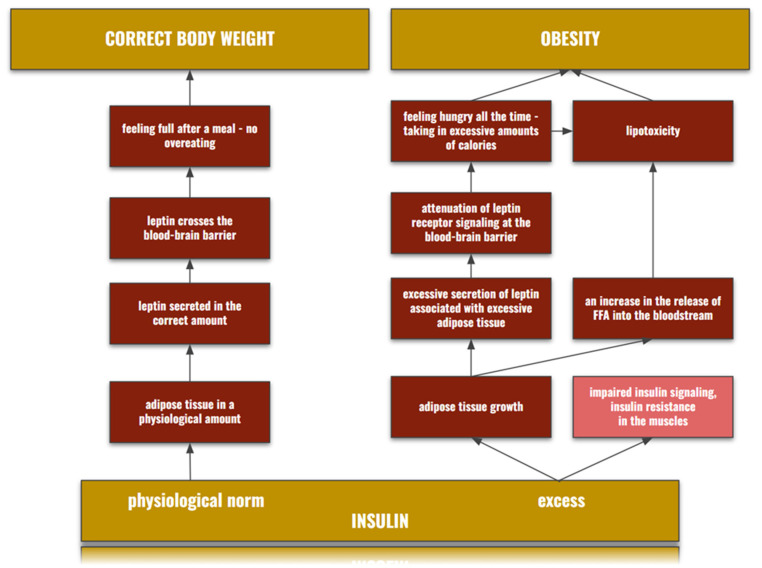
Influence of normal and excessive amounts of insulin on leptin level and development of obesity.

Bedir et al. [14] found that leptin may be the missing link between hyperuricemia and obesity. Leptin is a cytokine, the amount of which depends on the amount of adipose tissue in which it is synthesized. In addition, in smaller amounts, leptin is also produced in places such as muscles, the placenta, or the stomach—the more adipose tissue, the more leptin and the greater its release into the bloodstream. In the blood, it is found in two isoforms: bound to proteins and as a free form, a biologically active form [20,21]. Since its primary source is adipose tissue, the mechanism of leptin secretion is closely related to insulin and other peptide hormones of the pancreas, such as amylin or pancreatic polypeptides, by sending signals leading to a reduction in the amount of food intake [21]. Additionally, leptin expression is regulated by proinflammatory cytokines or glucocorticoids [22]. If the amount of adipose tissue is correct, leptin synthesis remains normal. When the amount of adipose tissue increases, the amount of inulin secreted in the body increases, which, driving the growth of adipose tissue, leads to an increase in the amount of leptin in the bloodstream [21]. It has also been proven that the consumption of high-fat duo increases the expression of proinflammatory cytokines, such as tumor necrosis alpha (TNF-α) or interleukin 6 (IL-6), causing the activation of proinflammatory pathways and leading to excessive secretion of leptin [20]. In the case of adipose tissue deficiency occurring during malnutrition, the amount of leptin in the body decreases [23]. The mechanisms of linking the amount of insulin with leptin have been presented in Figure 1.

Thus, it can be assumed that the increase in leptin secretion is proportional to the increase in body weight caused by the accumulation of adipose tissue and depends on the nutritional status [21].

In turn, ghrelin was first described as a growth hormone-releasing peptide in a rat’s stomach [24]. It regulates energy homeostasis, protecting the body against hunger, causing the desire to eat and reach for a meal, and regulating appetite control [24,25,26]. It is secreted mainly by the cells of the endocrine glands at the bottom of the stomach [26]. It exerts an orexigenic effect through the relationship with specific receptors of the hypothalamus, transmitting information about the nutritional status to the brain [24,25,27]. Food intake affects fat deposition and the release of growth hormones in the body. According to most studies, it inhibits insulin secretion by regulating glucose homeostasis. Unfortunately, it is also involved in the development of cancer and the formation of metastases [26]. Research suggests that postprandial ghrelin concentrations are lower in obese people, which confirms their feeling of lack of satiety. In addition, it has been proven that the level of ghrelin increases during weight loss [27]. Mention is also made of the pharmacological potential of therapy to inhibit ghrelin signaling in treating insulin resistance and type 2 diabetes [28].

Due to the ghrelin and leptin interaction, they are included in the “ghrelin-leptin tango”.

Despite demonstrating numerous relationships between leptin and UA, data about ghrelin and leptin levels in patients with urolithiasis are lacking in the existing literature. After entering the query “urolithiasis” and “ghrelin” in the Pubmed search engine, we did not find any results; after entering “urolithiasis” and “leptin,” we got only three [29,30,31]. For this reason, we decided to investigate what levels of ghrelin and leptin characterize the group with urolithiasis and whether they differ from those in healthy people.

The study aimed to assess the relationship between metabolic parameters, inflammation, UA, ghrelin, and leptin in patients with urolithiasis, hyperuricemia and MetS.

## 2. Materials and Methods

### 2.1. Study Groups

The studied group consisted of 60 patients (group termed urolithiasis patients—UP), which: (1) reported to the urology office and were qualified for the treatment due to the need for extracorporeal shockwave lithotripsy—(ESWL); (2) showed hyperuricemia (in men and post-menopausal women above 7 mg/dL (416.4 µmol/L), in premenopausal women above 6 mg/dL (356.9 µmol/L)) and did not take before any medications lowering UA; (3) had abnormal blood pressure values (>130/85 mmHg) or received antihypertensive treatment, and (4) showed an abnormal waist circumference > 94 cm in male(M), > 80 cm in female(F).

After the initial examination in the form of determining the level of serum glucose, high-density lipoprotein cholesterol (HDL-C), and triglycerides (TG) and performing morphology, samples were selected, leaving only those patients who showed at least 3 out of 5 features of MetS ((1) incorrect waist size—for Caucasian, Middle East, and Mediterranean population: 94 cm (M), 80 cm (F); (2) TG = 150 mg/dL or appropriate therapy; (3) High-density lipoprotein cholesterol (HDL-C) 40 mg/dL (M), 50 mg/dL (F), or appropriate therapy; (4) blood pressure: 130/85 mmHg or appropriate therapy, and (5) fasting blood glucose 100 mg/dL or using appropriate therapy) and did not show features of other acute or chronic inflammatory conditions. Finally, 57 patients aged 27–60 met the qualification criteria and were included in this study.

The control group (CG) comprised 30 healthy volunteers who reported to the laboratory for their check-ups. The conditions for participation in the study were age (18–60 years) and the absence of acute and chronic inflammation, pregnancy, and diagnosed diseases. After the initial examination in the form of determining the levels of glucose, HDL-C, and TG and performing morphology, samples were selected, leaving those patients who did not show signs of MetS and did not show signs of other acute or chronic inflammatory conditions. Finally, 29 patients aged 22–60 were qualified for the study.

**Figure 2:**
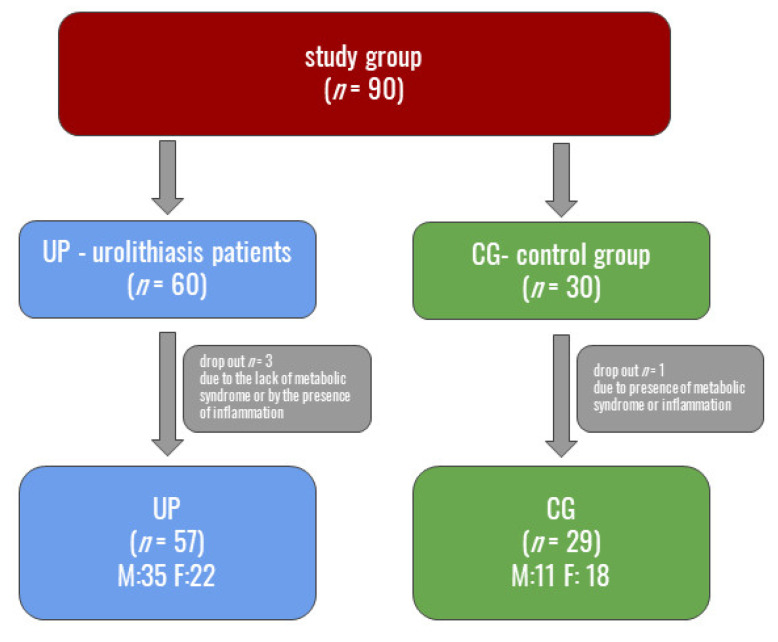
Division into groups selected for this study. F—female; M—male.

The structure of the study group is shown in Figure 2.

**Figure 3:**
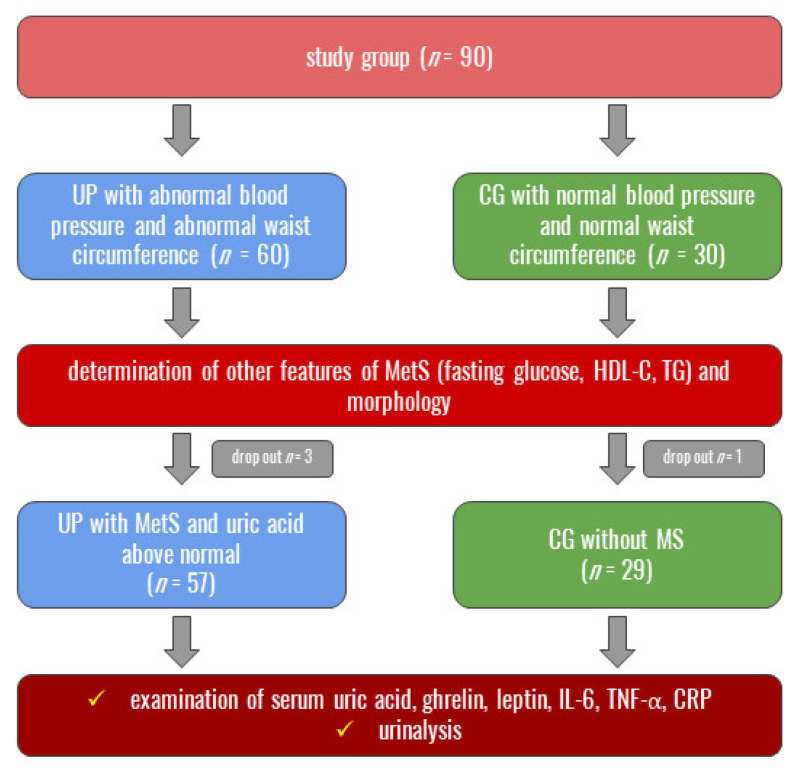
Model of performed tests. MetS—metabolic syndrome; HDL-C—high-density lipoprotein cholesterol; TG—triglycerides; IL-6—interleukin 6, TNF-α—tumor necrosis factor alpha, CRP–C-reactive protein.

After qualification, all eligible participants had their UA, ghrelin, leptin, IL-6, TNF-α, and CRP serum levels measured. Next, in both studied groups, urinalysis assessments were made. The full research model is presented in Figure 3.

### 2.2. Methods Used

All patients and healthy volunteers from the control group underwent a routine interview including personal data, age, weight and height (BMI calculation), waist circumference, blood pressure measurement, and signing the consent for the study.

In the next stage, blood was collected in the morning between 8.00 and 9.00 from the elbow joint in a total amount of about 9 mL into three test tubes:

- containing EDTA—performing morphology;
- without anticoagulant to obtain serum for other routine biochemical assays (creatinine, glucose (Glu), triglycerides (TG), high-density lipoprotein cholesterol (HDL-C)).

Serum was obtained after clotting (10 min) and centrifugation (10 min, 3000 RPM) of the collected blood. Then, they were distributed into Eppendorf tubes, frozen, and stored at −80 °C until assayed. All samples were subjected to the same procedure in order to eliminate factors that could have a significant impact on the determination results.

### 2.3. Determination of Serum Leptin, Ghrelin, IL-6, TNF-α and CRP

The concentration of the tested compounds was determined using SunRed tests using the Enzyme-Linked Immunosorbent Assay (ELISA) method using the TECAN-SUNRISE reader with the Magellan software. The kit uses monoclonal antibodies directed against the analyte’s antigenic determinants. The analyte to be determined is bound to solid phase-conjugated antibodies (placed on the surface of the wells), then biotinylated antibody against the compound to be determined and streptavidin-horseradish peroxidase conjugate (conjugate). The color reaction is obtained using tetramethylbenzidine (TMB). The absorbance of the sample was measured at a wavelength of 450 nm. The concentration of the parameters was read from the standard curve prepared from a series of dilutions of the standard of known concentration.

#### Urinalysis

Routine urinalysis was performed in the morning fasting state using dipstick tests.

### 2.4. Ethics

The study was conducted in Poland at the Poznan University of Medical Sciences (Department of Medical Chemistry and Laboratory Medicine) from February 2020 to August 2022. The study was conducted in accordance with the Declaration of Helsinki and approved by the Bioethics Committee of the Poznan University of Medical Science (No. 236/20/2020). Each patient gave informed written consent to participate in the study.

### 2.5. Statistical Analyses

Studied variables were defined using the median (median), minimum and maximum (minimum-maximum) values. Due to the nature of the variables, non-parametric tests were used for statistical analyses.

The relationship between the variables was assessed using the Mann–Whitney U test. If the test probability p exceeded the assumed significance level of alpha = 0.05, the distribution of variables in both groups was considered to be the same.

Correlations between measurable variables were checked using Spearman’s rank correlation coefficient significance test. If the test probability p exceeded the assumed significance level α = 0.05, it meant that there was no correlation between the tested variables.

*p*-value < 0.05 was considered statistically significant.

Statistical calculations were made using the STATISTICA 13.3 PL statistical package.

## 3. Results

### 3.1. Characteristics of the Study Group

The study involved 86 people, including 57 patients with urolithiasis and 29 healthy people in the control group. In the group of people with urolithiasis, there were 35 men and 22 women; all patients were characterized by arterial hypertension (>130/85 mmHg), abnormal waist circumference (>94 cm in men, 80 cm in women), abnormal UA value (in men and post-menopausal women above 7 mg/dL (416.4 µmol/L), in premenopausal women above 6 mg/dl (356.9 µmol/L)) and at least one of the other three features of MetS (abnormal glucose, HDL-C or TG). The control group included 11 men and 18 women without chronic diseases, showing up to two features of the metabolic syndrome. All subjects were aged 22–60. The characteristics of the surveyed persons are presented in Table 1.

Urine samples were tested in both groups. In the UP-average urine, specific gravity was 1.016 ± 0.007 g/mL. Protein in the urine was observed in 12 samples, with a mean value of 79.65 ± 88.16 mg/dL. Acidic urine (pH < 5.6) was observed in 38 patients, alkaline urine (pH > 7) in four patients, and pH = 6.5 in two patients. No glucose, ketones, bilirubin, or casts were observed in the urine samples. There were two patients with solitary UA crystals and one with solitary amorphous urates.

In the CG group, all the urinalysis results were within the normal limits, with pH = 6.5, specific gravity 1.019 ± 0.002 g/mL, and no glucose, ketones, bilirubin, or protein. Moreover, no crystals and casts were found in the urine sediment.

### 3.2. The Results of the Tested Parameters

The results of tested parameter determinations in the blood of the subjects have been presented in Table 2.

There were statistically significant differences in UA levels between the groups. Higher values than the control group characterized the group with urolithiasis. Moreover, statistically significant differences in ghrelin and leptin levels were observed. Lower ghrelin values and higher leptin values than the control group characterized the group with urolithiasis. The results of IL-6 in the group of patients with urolithiasis were significantly higher than in the control group. TNF-α in the group of patients with urolithiasis was significantly higher than those in the control group.

Next, the correlations between the studied parameters have been calculated.

The results of correlations between the examined parameters have been shown in Table 3 (the entire study group), Table 4 (the UP group), and Table 5 (the CG group).

We also checked whether individual parameters correlated with BMI categories. In the UP group, we have revealed that leptin concentration increased significantly with BMI if we divided BMI into three categories: normal weight (*n* = 17), overweight (*n* = 25), and obese (*n* = 15).

## 4. Discussion

UA concentrations in the blood of people with urolithiasis were found to be higher than in the control group and did not fall below the recommended reference values. This is an expected result due to the disease’s characteristics and the study group’s selection.

Our research showed a positive correlation between BMI and UA and a negative correlation with HDL-C. We found no correlation between BMI and WBC (white blood cells). Similar results were published by Zarrati et al. [32]; however, their studies also showed a significant correlation between BMI and TG levels. Moreover, in our research, BMI positively correlated with leptin and IL-6 levels, suggesting a link between visceral obesity and inflammatory process in the organism [33]. We also disclosed that leptin levels increased with BMI, which was confirmed by other studies [34,35]. Besides, we showed the expected inverse correlation between the parameters of the metabolic syndrome, such as HDL-C and glucose serum concentration, and between HDL-C and TG.

In the Brazilian longitudinal study of adult Health (ELSA—Brasil), serum UA equal to 5.0 mg/dL (297.4 µmol/L) was the best cutoff that discriminated incident MetS [36].

Researchers suggested that UA saturation in the urine rose in obese people [37,38,39,40]. High BMI has been specifically attributed to the formation of urinary stones since it has been confirmed that UA independently and positively correlates with the risk of obesity [39]. An increased incidence of urolithiasis of greater than 75% was observed in overweight and obese patients compared to their healthy counterparts [41,42].

Our research has shown that leptin levels were higher and ghrelin levels were lower in people with urolithiasis than in the control group. It is worth noting the existence of a positive correlation between leptin and UA. This confirms the thesis put forward by Bedir et al. and Fruehwald-Schultes et al. [14,43], that leptin can affect UA levels. Interestingly, in our subjects, in the case of ghrelin, we revealed a positive correlation only with leptin. There was no correlation of ghrelin with glucose or BMI, as in the study of Cruz-Dominguez et al. [44]; however, we observed that, among the UP study group, characterized by a higher BMI, ghrelin levels were lower. Studies by Nanjo et al. [40] also showed the correlations between ghrelin and UA, BMI, and TG. It would be worth conducting research on a larger number of people, specifying the degree of obesity, to check whether this relationship is confirmed or whether it is different in people with urolithiasis.

All subjects with urolithiasis and metabolic syndrome were characterized by significantly higher inflammatory markers IL-6 and TNF-α, as confirmed in the literature [45,46]. An elevation in serum UA, as that observed in cardio-metabolic disorders, may promote the expression of hepatic acute-phase reactants resulting in higher circulating levels of inflammatory biomarkers, as observed in our study among patients with MetS and urolithiasis.

The literature suggests oxidation of free fatty acids, systemic metabolic acidosis, and inhibition of lipoprotein lipase in adipose tissue as the mechanism of this phenomenon, which may lead to the development of insulin resistance. [45,46]. We also showed a link between leptin and proinflammatory parameters, such as IL-6 and TNF-α. This confirms the role of leptin in visceral obesity [33].

According to the results of our study, there is a powerful correlation between ghrelin and leptin levels and an average correlation between UA and leptin levels. This may indicate a significant association of diseases characterized by hyperuricemia, such as urolithiasis, with abnormal values of appetite hormones.

The study’s limitations include a small group of patients, which resulted from rigorous inclusion criteria, and the fact that the research was carried out during the COVID-19 pandemic. It should also be mentioned that the patients’ food diaries were not examined to determine their eating habits. In future studies, eating habits should be considered a crucial issue.

Significant age differences in the studied groups could impact the obtained results; however, they come from the assumed inclusion criteria—MetS and urolithiasis more often affect the elderly. It is challenging to find older people without associated ailments with MetS.

Moreover, we had no information on the type of stones; however, because the stones were radiolucent, primarily based on the patient’s available medical history and based on the attending doctors’ experiences, we could suggest that they were most likely formed from UA. This is quite a simplification, and it would be better if the study of the composition of the stones were carried out. However, such was the procedure in the ward that if the stones were large (and such were the case with all included patients, stones diameter exceeding 7 mm), the patient was immediately referred from the doctor’s office to the hospital for their removal. The limitation is that postoperative information was not collected.

This study results should be confirmed in subsequent studies, where eating habits would be carefully assessed and the composition of the stones checked.

Moreover, it would be worth examining additional groups in future research—people with high UA levels and MetS without urolithiasis and those without MetS. This would allow for other data to be compared.

The study’s strengths include that it was the first group of patients with urolithiasis to be examined for the levels of appetite hormones, such as ghrelin and leptin. We also showed that, unlike leptin, ghrelin correlates with other outcomes such as IL-6, TNF-α, and UA only in the group of people with urolithiasis which may be helpful in further research on appetite hormones in patients with this condition.

## 5. Conclusions

Despite some limitations, this study revealed significant correlations between ghrelin and leptin, between these hormones and IL-6, and between leptin and UA in patients with hyperuricemia, urolithiasis and MetS. In addition, we confirmed that hyperuricemia is associated with some components of MetS. We also found that leptin concentrations in studied patients arose with increasing BMI, if BMI was divided into categories. Proving the link between obesity and many diseases, including urolithiasis, may help to treat this disease better. These findings open a new path to broader research on appetite hormones in people with urolithiasis. Due to the diet-dependent nature of the disease, which is urolithiasis, this topic should be looked at more closely, as research on appetite hormones, such as ghrelin and leptin, may positively affect the dietary aspects of the treatment of this disease.

## Figures and Tables

**Table 1:** Baseline characteristics of the participants.

**Variable**	**Study Group**		*p* *
	**Urolithiasis Patients (UP)**	**Control Group (CG)**	
	***n* = 57** **%M: 54.38** **(*n* = 31)**	***n* = 29** **%M: 37.93** **(*n* = 11)**	
	Mean ± SD Median (Min–Max)		
Age [years]	59.6 ± 8.2 52 (27–60)	32.1 ± 8.2 29 (22–51)	<0.001
BMI [kg/m^2^]	26.8 ± 4.2 26.7 (18.6–36.5)	22.6 ± 3.3 22.3 (17.9–28.4)	<0.001
Glu [mg/dL]	109.1 ± 22.4 109 (83.6–188.8)	91.4 ± 5.6 91.9 (74.7–98.4)	<0.001
TG [mg/dL]	125.5 ± 51.0 125.53 (13–351)	100.3 ± 45.8 89 (47–217)	0.010
HDL-C [mg/dL]	49.3 ± 14.3 47 (23–88)	58.8 ± 12.5 55 (41–92)	0.002
eGFR [mL/min/1.73 m^2^] based on the MDRD study equation ^%^	86.9 ± 10.83 87 (71–120)	89.4 ± 10.4 89 (86–114)	0.171
CRP [mg/L]	3.95 ± 2.51 3.3 (0.81–8.71)	2.00 ± 1.20 1.79 (0.51–4.81)	0.005
*n* (%) **			
the presence of the metabolic syndrome	24 (100%)	0	<0.001

M—male. * *p* was calculated to evaluate the differences between groups concerning selected variables. ^%^—estimated GFR (eGFR), according to the KDIGO 2012 recommendation, was calculated based on the Modification of Diet in Renal Disease (MDRD) formula: eGFR = 186 × (creatinine concentration [mg/dL]) − 1.154 × (age) − 0.203 × (0.724) for female. ** *n* (%)—the number of participants and their percentage content in the studied groups.

**Table 2:** Average results of tested parameter concentrations in the study group.

**Parameter**	**Study Group (*n* = 53)**		*p* *
	**UP (*n* = 57)**	**CG (*n* = 29)**	
	Mean ± SD Median (Min–Max)		
UA [mg/dL] [µmol/L]	7.995 ± 0.911 8.098 (6.321–9.900) 475.543 ± 54.186 481.669 (375.973–588.852)	4.717 ± 0.8931 4.570 (3.020 – 6.500) 280.567 ± 53.122 271.824 (179.630–386.620)	<0.001
Leptin [ng/mL]	13.872 ± 9.368 10.783 (5.483–43.458)	9.693 ± 3.0183 9.093 (4.887 – 18.251)	0.045
Ghrelin [pg/mL]	1216.674 ± 1384.743 779.717 (297.870–9054.00)	1597.430 ± 921.1917 1464.910 (587.108 – 4803.72)	<0.001
IL-6 [pg/mL]	8.739 ± 5.815 8.090 (1.023–22.123)	2.631 ± 4.011 3.0.15 (0.131–6.091)	<0.001
TNF-α [ng/L]	151.128 ± 67.379 142.675 (45.987–451.230)	84.697 ± 98.398 72.431 (32.499 – 203.480)	<0.001

* *p* was calculated to evaluate the differences between groups concerning selected variables.

**Table 3:** The most important significant correlations between the examined parameters in the entire study group.

Parameter	BMI	Glu	UA	TG	HDL-C	Leptin	IL-6	Ghrelin	TNF-α	CRP
**BMI**	-		r = 0.417 *p* < 0.001		r = −0.264 *p* = 0.018	r = 0.234 *p* = 0.037	r = 0.377 *p* = 0.001			r = 0.276 *p* = 0.036
**Glu**		-	r = 0.482 *p* < 0.001		r = −0.240 *p* = 0.32	r = 0.2878 *p* = 0.010	r = 0.367 *p* = 0.001		r = 0.390 *p* < 0.001	
**UA**	r = 0.417 *p* < 0.001	r = 0.482 *p* < 0.001	-	r = 0.295 *p* = 0.008		r = 0.380 *p* < 0.001	r = 0.606 *p* < 0.001		r = 0.460 *p* < 0.001	
**TG**			r = 0.295 *p* = 0.008	-	r = -0.506 *p* < 0.001	r = 0.267 *p* = 0.017				
**HDL-C**	r = −0.264 *p* = 0.018	r = -0.240 *p* = 0.32		r = −0.506 *p* < 0.001	-					
**Leptin**	r = 0.234 *p* = 0.037	r = 0.288 *p* = 0.010	r = 0.380 *p* < 0.001	r = 0.267 *p* = 0.017		-	r = 0.6618 *p* < 0.001	r = 0.620 *p* < 0.001	r = 0.248 *p* = 0.027	
**IL-6**	r = 0.377 *p* = 0.001	r = 0.367 *p* = 0.001	r = 0.606 *p* < 0.001			r = 0.662 *p* < 0.001	-		r = 0.436 *p* < 0.001	
**Ghrelin**						r = 0.620 *p* < 0.001		-		
**TNF-α**		r = 0.390 *p* < 0.001	r = 0.4600 *p* < 0.001			r = 0.248 *p* = 0.027	r = 0.436 *p* < 0.001		-	
**CRP**	r = 0.276 *p* = 0.036									-

**Table 4:** Statistically significant correlations between the tested parameters in the UP group.

Parameter	IL-6	Ghrelin	Leptin	UA	CRP	BMI
**IL-6**	-	r = 0.370 *p* = 0.007	r = 0.648 *p* < 0.001	r = 0.481 *p* < 0.001		
**Ghrelin**	r = 0.370 *p* = 0.007	-	r = 0.831 *p* < 0.001	-		
**Leptin**	r = 0.648 *p* < 0.001	r = 0.831 *p* < 0.001	-	r = 0.400 *p* = 0.004		
**UA**	r = 0.481 *p* < 0.001	-	r = 0.400 *p* = 0.004	-		
**CRP**					-	r = 0.276 *p* = 0.038
**BMI**					r = 0.276 *p* = 0.038	-

**Table 5:** Statistically significant correlations between the examined parameters in CG group.

Parameter	IL-6	Ghrelin	Leptin
**IL-6**	-		r = 0.411 *p* = 0.27
**Ghrelin**		-	r = 0.487 *p* = 0.007
**Leptin**	r = 0.411 *p* = 0.270	r = 0.487 *p* = 0.007	-

## Data Availability

All necessary data are included in the paper.
